# A Novel Digital Self-management Intervention for Symptoms of Fatigue, Pain, and Urgency in Inflammatory Bowel Disease: Describing the Process of Development

**DOI:** 10.2196/33001

**Published:** 2022-05-18

**Authors:** Louise Sweeney, Sula Windgassen, Micol Artom, Christine Norton, Sophie Fawson, Rona Moss-Morris

**Affiliations:** 1 Health Psychology Section King's College London London United Kingdom; 2 NHS Digital London United Kingdom

**Keywords:** inflammatory bowel disease, symptoms, self-management, intervention development, digital health

## Abstract

**Background:**

Empirical studies and systematic reviews have demonstrated the role of biological, cognitive, behavioral, and emotional factors in fatigue, pain, and urgency in inflammatory bowel disease (IBD). Behavioral management that addresses the cognitive, behavioral, and emotional factors offered alongside medical treatment is seldom available to people with IBD. Digital interventions provide a potentially scalable and cost-effective way of providing behavioral support to patients.

**Objective:**

This paper aimed to describe the process of developing a supported digital self-management intervention for fatigue, pain, and urgency in IBD using theory and evidence-based approaches and stakeholder input.

**Methods:**

The Medical Research Council framework for complex health interventions and a person-based approach were used to guide intervention development, consulting with 87 patients with IBD and 60 nurses. These frameworks informed the selection and use of a theoretical model that subsequently guided cognitive behaviorally based intervention content. They also guided the design of tailored digital intervention pathways for individuals with IBD that matched the predominant symptoms.

**Results:**

A transsymptomatic cognitive behavioral framework of symptom perpetuation was developed for the symptoms of fatigue, pain, and urgency in IBD. A logic model was used to define the intervention techniques. Patient feedback and qualitative interviews refined the website content and functionalities, including the use of visual aids, email reminders, and graphical tracking of symptoms. Nurse focus groups informed the volume and delivery model of the therapist *facilitator* support. Ratings of acceptability out of 10 following feasibility testing (31/87, 36%) demonstrated accessibility (scoring 9.43, SD 1.040), ease (scoring 8.07, SD 3.205), clarity, and the relevant tone of the intervention. The final intervention comprised 12 web-based sessions (8 core and 4 symptom-specific), with one 30-minute facilitator phone call following session 1 and subsequent on-site messaging.

**Conclusions:**

The use of theory and integration of stakeholders’ views throughout informed the development of an evidence-based digital intervention for fatigue, pain, and urgency in IBD. This is the first web-based self-management intervention designed to address these multiple symptoms with the aim of improving the quality of life and reducing the symptom burden of IBD. The intervention is being tested in a large multicenter randomized controlled trial.

**Trial Registration:**

ISRCTN Registry ISRCTN71618461; https://www.isrctn.com/ISRCTN71618461

## Introduction

### Background

Inflammatory bowel disease (IBD) refers to both Crohn disease and ulcerative colitis, which are chronic relapsing–remitting inflammatory disorders of the digestive tract. IBD has a growing prevalence [1], with an estimated 5 million people worldwide and between 2.5 and 3 million people in Europe alone diagnosed with IBD [1,2]. Periods of severe disease flare-ups can involve hospitalization and surgery [3,4]. During remission, it is common for people with IBD to continue to experience symptoms. The most commonly reported symptoms include fatigue, urgency and/or incontinence, and pain [5]. Fatigue is widely reported to have a disruptive impact on everyday functioning and quality of life (QoL) in IBD [6,7]. Similarly, urgency of defecation with or without frank fecal incontinence [8] and pain [9] are consistently cited as particularly burdensome symptoms. In a research priority setting exercise, both patients and clinicians have highlighted a better understanding and management of these symptoms [10].

Currently, the medical management of IBD alone does not always adequately treat these symptoms [11]. There is an observable disconnect between symptoms and the degree of gut inflammation in IBD, with evidence showing that even when disease activity is low, people can experience ongoing fatigue, pain, and urgency [12-14]. Research has demonstrated the complex, multidimensional, and multifactorial pathogenesis of these symptoms. Extensive evidence demonstrates the interactive role of specific cognitive, behavioral, and emotional factors in the experience of fatigue, pain, and urgency/incontinence in IBD [7,15,16]. These factors also affect the QoL of patients with IBD.

### Cognitive Behavioral Therapy for Gastrointestinal Conditions

There is substantial symptom overlap among irritable bowel syndrome (IBS), another gastrointestinal condition, and IBD [17]. Individuals with IBS experience bowel-related abdominal pain and, often, urgency and fatigue. Cognitive behavioral therapy (CBT) is the most extensively researched psychological treatment approach, demonstrating efficacy in reducing symptom severity and enhancing QoL in patients with IBS [18-20]. CBT targets IBS-related concerns and introduces other psychological techniques such as relaxation and mindfulness to interrupt the vicious cycles of symptoms and psychological processes that perpetuate symptoms [18]. Preliminary research has demonstrated the feasibility of improving fatigue and pain using CBT in IBD [21,22]. This provides a rationale for the use of CBT in targeting fatigue, pain, and urgency in patients with IBD in remission. To date, most psychological interventions for IBD have been designed to reduce affective outcomes (ie, anxiety or depression) rather than targeting other symptoms that are burdensome for patients [23,24].

Engaging patients in their health-related decisions can result in better disease and QoL-related outcomes [24,25]. Web-based self-management resources provide a means for patients to be more involved with their care, with greater scope for accessing information and engaging with their care pathways [26]. However, attrition is recognized as a significant issue in IBD web-based intervention studies, with several contributing factors, including a lack of direct contact with a health care professional [27]. A European consensus statement [28] indicated that IBD nurses might be well placed to facilitate self-management and psychological support for patients experiencing fatigue, pain, and incontinence. As such, combining web-based programs with IBD nurses acting as intervention *facilitators* may empower patients while reducing attrition rates and increasing the effectiveness and cost-effectiveness of an intervention [29].

### Theory-Driven Intervention Development

To develop a theory-driven and evidence-based intervention with maximum opportunity for wider implementation, it is necessary to draw upon intervention development frameworks. A recently published taxonomy of approaches to developing interventions to improve health suggests that there are 2 key ways of designing and creating an intervention, one of which prioritizes working with the target population and the other which focuses on theory [30]. The 2008 Medical Research Council (MRC) guidance on the development of complex interventions is a framework guiding the use of theory to inform intervention development [31]. This process is iterative, including the identification of the evidence base, systematic development of theory, modeling processes and outcomes, and feasibility and pilot testing in the development stage. The *person-based approach* is an intervention development framework that prioritizes input and guidance from the target population [32]. This uses in-depth qualitative approaches to understand the behavioral aspects of user engagement with interventions. It addresses user-centered design and feedback as an integral aspect of intervention development. This enables developers to summarize design features that are likely to be important, appealing, and persuasive for intended users to guide principles for intervention development.

Currently, there is no guidance on how to integrate both theory-based and target population–driven approaches. Normalization process theory (NPT) is an approach that focuses on factors that are likely to facilitate and inhibit the implementation of complex interventions into practice [33,34]. It recommends consulting with the target population and key stakeholders to ensure the acceptability and ease of implementation of the intervention. For example, a lack of adequate consultation with health care professionals can result in design flaws that reduce health care professionals’ readiness to support the intervention and its applicability and feasibility within health care settings [35]. The involvement of end users in the development of web-based interventions in IBD outpatient settings can reduce resistance and other barriers to adoption [25,36]. Methods available for gathering input from the target population within a person-based approach include (1) using stakeholders as research participants, where data (quantitative and/or qualitative) are drawn from participants and inductively analyzed by researchers, and (2) involving stakeholders as research partners in patient and public involvement (PPI) activity, where they actively direct and inform the research processes [37].

### Aims of the Paper

The aim of this paper is to describe the use of the MRC complex intervention framework alongside an NPT-guided person-centered approach to develop a theory and empirically driven facilitator-supported, tailored, digital cognitive behavioral intervention for fatigue, pain, and urgency/incontinence in IBD called IBD-BOOST.

## Methods

### Overview

**Figure 1:**
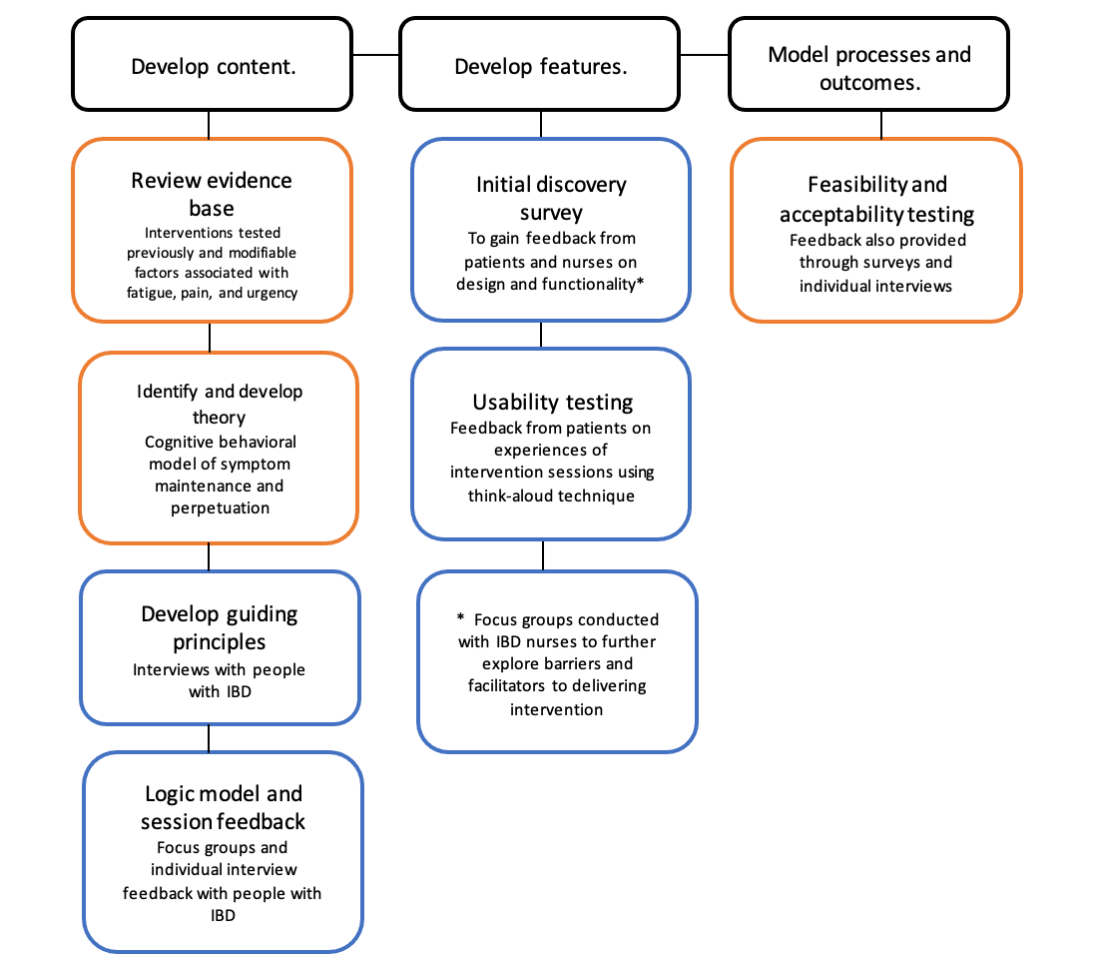
Intervention development stages as recommended by the Medical Research Council guidance (orange) and person-based approach (blue). IBD: inflammatory bowel disease.

The 2008 MRC guidance (the most recent, available guidance at the time we began the project) [31] and the person-based approach to intervention development [32] were combined into 3 areas to inform the intervention development sequence, as depicted in Figure 1. These areas of development were not sequential but iterative, allowing for amendments and updates throughout the intervention development process.

### Stakeholder Engagement

We consulted key stakeholders (patients and nurses) during the qualitative interviews and PPI activities. Qualitative interviews were conducted in 5 focus groups and in individual interviews with people with IBD to explore experiences of the 3 symptoms and the thoughts of people with IBD on a web-based intervention for fatigue, pain, and urgency/incontinence. These qualitative findings have been reported in full elsewhere [38,39].

PPI activity included consultations with 87 people with IBD and 60 IBD nurses participating in PPI. Some members of the PPI bank were approached multiple times for feedback and testing purposes. A summary of the participants in each type of PPI activity across each development phase is depicted in Table 1.

**Table 1:** Summary of stakeholder involvement at each intervention development stage^a^.

Intervention development stage		People with IBD^b^ (n=87), n (%)	Nurses (n=60), n (%)
Patient survey to gather initial insight on functionality and design of the intervention		56 (64)	—^c^
Patient survey on intervention name options		20 (23)	—
Patient surveys (thrice) on the web-based intervention logo		20 (23)	—
Patient survey on illustrations and character names		14 (16)	—
Initial discovery interviews to gather key characteristics of the target users		5 (6)	—
Focus groups (twice) on the intervention logic model and session overview		4 (4)	—
**Intervention paper session content feedback questionnaires**			
	Sessions 1-3	5 (6)	—
	Sessions 4-6	4 (4)	—
	Symptom-specific or summary session	3 (3)	—
Session 1 usability testing think-aloud interviews		10 (11)	—
8-week patient feasibility testing of the entire program		31 (36)	—
My Tasks page usability testing think-aloud interview		4 (4)	—
IBD nurse survey on training and resource needs to support the intervention		—	45 (75)
IBD nurse focus groups to gather views on intervention support and training needs		—	60 (100)

^a^Some stakeholders took part in >1 activity.

^b^IBD: inflammatory bowel disease.

^c^Patient and public involvement activity for people with IBD only.

### Developing Content

#### Identifying the Evidence Base for Potentially Modifiable Cognitive Behavioral Factors

The MRC framework [31] recommends identifying the relevant evidence base for an intervention. Systematic reviews of nonpharmacological interventions for fatigue, pain, and urgency/incontinence in IBD were completed by members of the team before developing the intervention. These informed the nature of the problem (symptoms of fatigue, pain, and urgency/incontinence), their causes, and the key modifiable factors with the greatest scope for change. Papers identifying cognitive, behavioral, and emotional factors related to symptoms were collected to inform the development of a CBT-based intervention. Gaps in the literature were also identified and addressed in subsequent empirical studies designed to explore and develop a conceptual understanding of the symptoms individually.

#### Identifying Theory and Components of the Intervention

Theoretical models used in interventions for fatigue, pain, and urgency/incontinence in related long-term conditions were identified. An overarching cognitive behavioral model was created transsymptomatically (for fatigue, pain, and urgency). This entailed (1) theoretical development to incorporate distinct and overlapping cognitive, behavioral, and emotional factors associated with symptoms; (2) development of an intervention logic model to identify treatment targets; and (3) consulting treatment protocols previously shown to be effective for symptom management in other long-term conditions.

#### Developing Guiding Principles

Interviews were conducted with PPI members (people with IBD) to inform initial intervention planning and gather views on the intervention content elements. A total of 5 interviews were conducted with people with IBD to develop *guiding principles* for the intervention. This informed us of what needs were being addressed and what contextual factors the intervention should take into account (illness experience and access to care).

### Developing a Logic Model and Session Feedback

An outline for the intervention was created by combining the findings from theory and user consultation, as described in the 2 previous sections. The PPI groups then provided feedback on the logic model for the intervention and session plans. Of the 87 patients, 5 (6%) people gave feedback on sessions 1 to 3, and 4 (5%) gave feedback on sessions 4 to 6, whereas 3 (4%) gave feedback on the symptom-specific and summary sessions. Individuals were provided with a paper or electronic document version of their assigned sessions and asked to read through the content and exercises and provide feedback through a feedback form before returning to the study team. The feedback was used to inform adaptations of the intervention structure and content.

### Developing Features

The person-based approach [32] was also used to inform the development and refinement of the features of the web-based intervention to aid uptake and meet the needs of the patients. Approximately 64% (56/87) of people with IBD and 75% (45/60) of IBD nurses responded to an initial survey to inform intervention functionality and design elements, such as the program name and logo, as well as the intervention format. Items included “how much time per week would you be able to spend on the programme?” and “if you were to give a name [label] to the tasks to complete between sessions, what would it be?” Focus groups were conducted with nurses to better understand contextual factors, such as barriers and facilitators related to supporting patients in the intervention, to optimize the implementation of the intervention as recommended by the NPT.

Once the draft intervention sessions had been developed, usability testing think-aloud interviews were conducted to collect feedback on usability, function, and perceptions of intervention content. This is where verbalized thoughts from users were provided while they interacted with an interface and its features [40]. A total of 10 think-aloud usability testing interviews were conducted to understand the experience of using session 1 for people with IBD.

### Modeling Processes and Outcomes: Testing the Feasibility and Acceptability of the Intervention

The MRC guidance identifies *modeling processes and outcomes* as a key part of intervention development. This involves testing the feasibility and acceptability of the intervention. Guidelines on how to model processes and outcomes are limited [41]. This step was aimed at assessing whether and how the intervention functions to deliver the desired outcomes. As this intervention was developed as part of a program grant, the substantive part of the assessment of processes and outcomes came after the intervention was developed and is being assessed in multiple stages [22,42]. However, initial assessments of the feasibility of the intervention were included in the preliminary intervention development process. This included people with IBD (31/87, 36%) who were given access to the entire intervention for a period of 8 weeks and who provided feedback through web-based surveys or telephone calls after 1week (30/31, 97%), 4 weeks (24/31, 77%), and 8 weeks (21/31, 68%; decrease because of dropout at follow-up). People were asked to give feedback after using the intervention alone so that their experience would not be influenced by the presence of a researcher [43].

### Ethics Approval

The study was carried out in accordance with the 18th World Assembly, Helsinki 1964, including later revisions and other relevant ethical guidance, which provide recommendations for physicians involved in human subjects research. IBD-BOOST obtained ethical approval (19/LO/0750) from a recognized National Research Ethics Service Committee and Health Research Authority.

## Results

### Stakeholder Engagement

Stakeholder input was used iteratively at multiple stages during intervention development. Therefore, it is detailed in the subsequent sections.

### Developing Content

#### Review of Nonpharmacological Interventions

A review of the literature on nonpharmacological interventions for fatigue, pain, and urgency in IBD demonstrated a lack of theoretically grounded interventions with demonstrable efficacy for improving these outcomes. A Cochrane review included only 5 nonpharmacological interventions for IBD fatigue [44], including electroacupuncture, CBT, solution-focused therapy, and advice on physical activity. The studies were rated as low quality. There was a similarly limited and heterogeneous pool of studies with small sample sizes for IBD pain [45]. For fecal incontinence, no studies have directly tested a psychological intervention in IBD; however, the limited evidence available suggests that once active disease and differential diagnoses have been ruled out, individualized management for each patient targeted at improving QoL is recommended [46].

#### Relevant Theory and Associated Empirical Evidence

A cognitive behavioral model of symptom perpetuation was identified as a framework from which to understand and create changes across fatigue, pain, and urgency. The cognitive behavioral model postulates that the way individuals think about and perceive their experiences (symptoms) affects how they feel and consequently respond to them [47]. Interventions that target unhelpful thoughts about symptoms and unhelpful behavioral responses have the potential to improve distress arising from symptoms and improve the symptoms themselves [48]. Although physiological triggers may differ, similar affective, cognitive, and behavioral responses to symptoms appear to exacerbate and maintain symptoms across long-term conditions [49]. A number of systematic reviews have identified specific cognitive, behavioral, and emotional factors associated with fatigue, pain, and urgency, along with other psychological factors [7,15,16,50,51]. Anxiety and depression were found to be associated with each symptom independent of disease activity.

Common cognitive factors associated with fatigue, pain, and urgency include negative perceptions of symptoms and catastrophizing [52-54]. Behavioral factors shared across the 3 symptoms included avoidance of activity, generally because of anxiety about outcomes specific to the symptoms [53,55-57]. Boom-bust patterns of behavior, which are common in other long-term conditions, including IBS [58,59], were also identified in studies exploring IBD pain and fatigue [52,53,55,56,60]. In IBD, pain, acceptance, pain self-efficacy, and mental well-being are associated with lower pain severity and pain-related disability [53,56]. A range of safety-seeking and avoidance behaviors designed to avert the possibility of incontinence was identified as often having a significant cognitive and affective burden [8,16,61,62].

### Developing a Logic Model and Session Feedback

#### Overview

**Figure 2:**
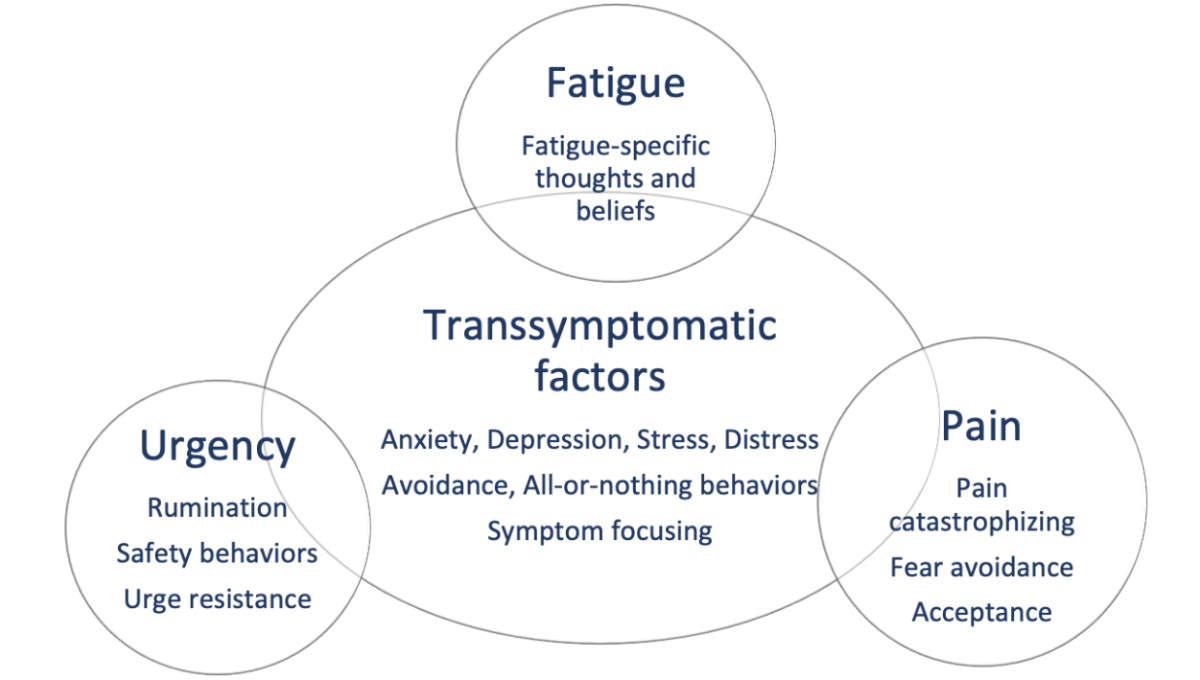
Example of overlap and symptom-specific modifiable factors in inflammatory bowel disease identified from the evidence base (systematic reviews and empirical evidence).

**Figure 3:**
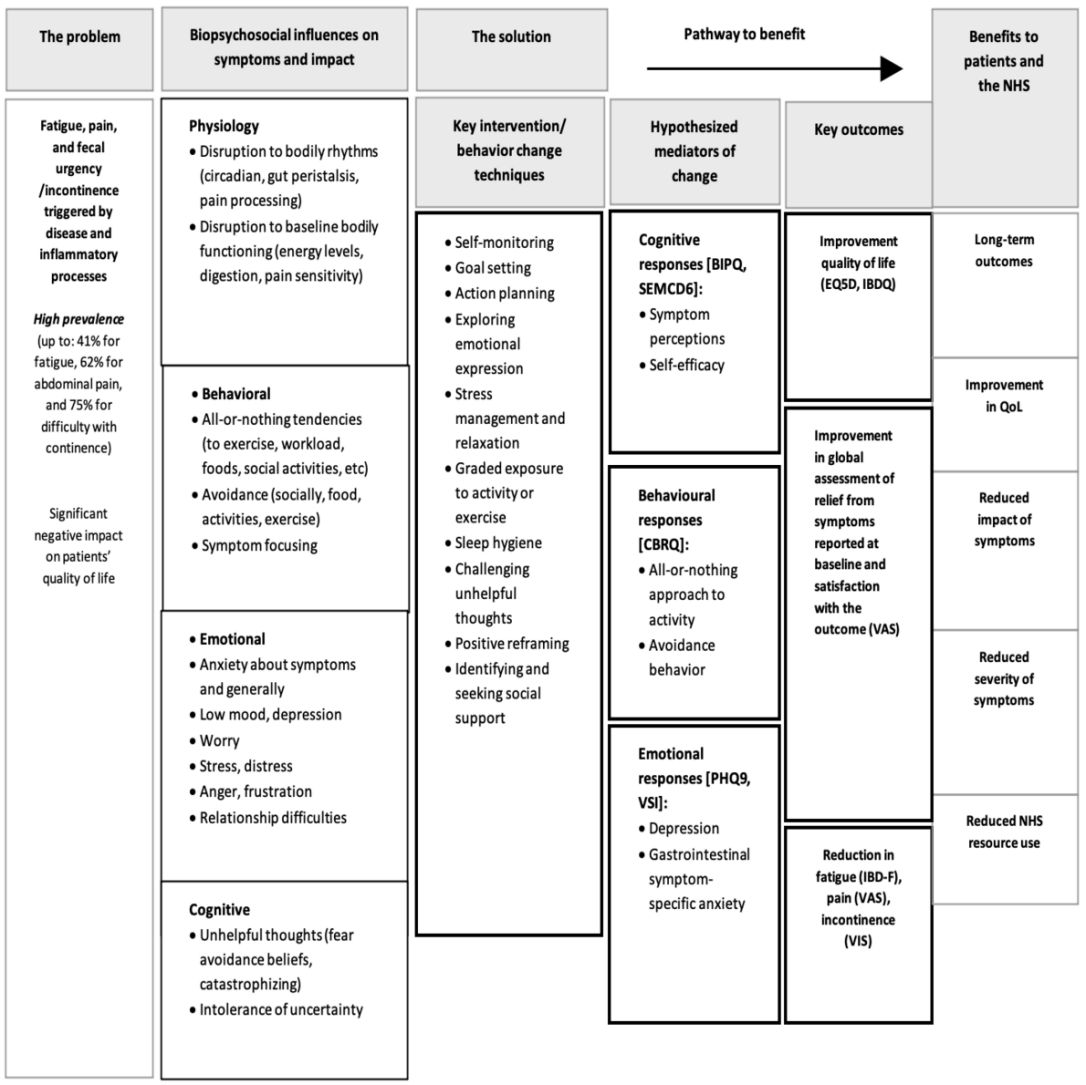
BOOST web-based self-management program logic model. BIPQ: Brief Illness Perception Questionnaire; CBRQ: Cognitive and Behavioral Responses Questionnaire; EQ5D: European Quality of Life Scale 5 Dimension Scale; IBD-F: Inflammatory Bowel Disease-Fatigue; IBDQ: Inflammatory Bowel Disease Questionnaire; NHS: National Health Service; PHQ-9: Patient Health Questionnaire-9; QoL: quality of life; SEMCD6: Self-Efficacy for Managing Chronic Disease 6-item Scale; VAS: Visual Analog Scale; VIS: Vaizey Incontinence Score; VSI: Visceral Sensitivity Index.

The overlapping and distinct psychological factors associated with symptoms of fatigue, pain, and urgency in IBD were summarized and informed a draft of an intervention logic model (Figure 2) and a protocol of intervention sessions. Intervention techniques used in cognitive behavioral interventions were mapped onto the identified psychosocial factors (Figure 3). For example, graded activity and goal-setting techniques were used to target avoidant and all-or-nothing behaviors. Stress management techniques and identifying or challenging thoughts through diary monitoring were applied to reduce distress and unhelpful thoughts (catastrophizing and fear avoidance), respectively. A cognitive behavioral model of symptom perpetuation has been applied in interventions for chronic symptom management in other long-term conditions [63-65]. Consequently, protocols and manuals used for these prior interventions were consulted (with the authors’ permission) to provide preliminary guidance on the session structure and format.

Although cognitive behavioral techniques were applied to target psychosocial factors relevant across symptoms, it was critical to understand the key distinctions and influential factors for specific symptoms. For example, the likelihood of incontinence is higher than that in other gastrointestinal conditions such as IBS *because of* factors such as internal and external sphincter defects, surgery (eg, anal fistula), and loose stool [8,16,66,67]. Therefore, targeting *safety behaviors* and avoidance in IBD, as is commonly done in CBT for IBS [19], required a tailored approach appropriate for the degree of incontinence experienced in IBD. Furthermore, behavioral strategies to improve incontinence may include exercises such as pelvic floor and sphincter exercises designed to improve muscle function and increase bowel control [68]. This further supports the rationale for symptom-specific content and general content applicable across symptoms.

#### Patient-Identified Intervention Development Needs

From the 5 interviews conducted to develop *guiding principles*, newly diagnosed people reported inconsistent access to clear and reliable sources of information on IBD and barriers to finding guidance that matched their unique experience of symptoms. People with a longer time since diagnosis expressed the need to better track their symptoms over time to identify patterns and better understand the impact of psychological factors on symptoms. All interviewees voiced the need to confront their experience with others’ experience of IBD and find ways in which to feel more socially supported and validated in relation to their symptoms. Dibley et al [39] further explored intervention needs for people with IBD in qualitative research focus groups, and the findings are described elsewhere

#### Logic Model and Feedback on Content in Paper Format

PPI participants (4/87, 4%) found the model (Figure 2) clear and comprehensive and could relate to the behaviors, thoughts, and emotions represented. They thought that all topics covered were valuable; however, unsurprisingly, people differed in which topics they perceived as more or less salient. Diet was identified as a missing topic and was therefore added to session 2. Participants expressed contrasting opinions on whether to make all sessions compulsory or allow the choice of which sessions to do. It was decided to make the core sessions compulsory to ensure an equal level of knowledge of the key topics for everyone in the program, with a choice of symptom-specific sessions.

Individuals reported the sessions as very helpful. They found the information accessible and concise but also comprehensive and informative. Interactive activities, patient stories, and quotes were identified as the most useful. The language was described as friendly, positive, and encouraging. However, participants suggested the need to reduce medical terms and simplify the language used in specific sections, such as those about the central nervous system and pain transmission.

### Developing Features

#### Surveys on Design and Functionality

The results from the survey (n=101) indicated several desirable functionalities (summarized in Textbox 1), such as a preference for completing the program on a mobile phone compared with a computer or tablet. People reported wanting the ability to complete the intervention in bite-sized chunks of time rather than in long sessions. Similarly, most suggested that they would be able to spend ≤60 minutes per week on the program. Key findings were the preferences of people with IBD to receive facilitator support via email or web-based messages over telephone and to conduct support sessions in the evening rather than in the daytime. By contrast, IBD nurses preferred to conduct support sessions during the day. These discrepancies further supported the use of focus groups with nurses to understand barriers and facilitators in supporting patients in the intervention (Textbox 1).

**Desirable content and functionalities incorporated and not incorporated in intervention and reasons why if not included.**

**Content and functionality incorporated into intervention**

- Interactive diagrams and aids
- Reminders to log in and complete tasks
- Bookmark pages
- Videos
- Links to external resources
- Notepad
- Personal calendar
- Graphical symptom tracker
- Content around diet in irritable bowel syndrome (a small section on diet included in the activity or exercise session but did not lead to separate sessions, given the complexity of diet in irritable bowel syndrome and inconclusive evidence)

**Content and functionality desired but not incorporated**

- Mobile app (however, there is an ability to access BOOST website via mobile or tablet through a browser). Reason: The program was mobile phone optimized (ie, easy to few and for use on a mobile phone), but the cost of a mobile app was out of budget
- Discussion forum with other users. Reason: Time required to moderate discussion and confidentiality

*BOOST* was the most popular name for the program as people found it positive, optimistic, and supportive. The preference for the logo with an arrow pointing forward was because of its association with gradually becoming better and the lack of medical reference, which people found anxiety provoking. From the illustrations presented for the characters in the program, participants selected the most gender- and age-neutral ones. When given a choice of names, participants chose *Sam* and *Alex*. To ensure inclusivity and diversity, the name *Ali* was also chosen. An explanatory note about being inclusive and avoiding bias toward a particular gender was included at the beginning of the program.

#### Usability Testing Think-Aloud Interviews

People with IBD (10/87, 11%) liked the ability to take a break during the sessions, the short paragraphs of text on each page, and the video summary of the session content. By contrast, people struggled to see links on the pages, found it difficult to go back to the home page, and wanted the quotes to stand out more from the rest of the text. People also reported that some sessions were too text heavy. As a result, the color of the links was changed, a home button was added, and quotes were formatted with a yellow background. Optional text sections were added to allow people to minimize or expand the amount of text to read.

#### Nurse Focus Groups

A total of 5 focus groups with 75% (45/60) of the nurses were conducted to understand the contextual factors that may influence the long-term implementation of the intervention. The use of *facilitator* was chosen over *therapist* as nurses were seen as taking a supportive rather than central role in the individual’s participation in the program. The focus groups revealed the limited time and resources available to nurses for multiple phone calls and concerns regarding lack of training. Consequently, the intensity of facilitator support was modified from four to one 30-minute phone call, and a comprehensive facilitator training plan was developed. The results of these focus groups were analyzed using thematic analysis and are presented elsewhere [69].

### Finalized Intervention

Following the stages of developing content and features, a complete intervention was finalized to be tested for feasibility and acceptability.

#### Program Content

The BOOST content comprises education and behavioral, cognitive, and emotional techniques. The individual is supported in developing a multifactorial understanding of the symptoms of pain, fatigue, and urgency that considers the triggers and maintenance factors. Participants would complete interactive assessments to create their own model of symptoms (vicious cycle) to identify possible behavioral, cognitive, and emotional factors that perpetuate their most distressing symptoms. The participants’ vicious cycle was used as a rationale to implement behavioral, cognitive, and lifestyle changes targeted at reducing the impact of precipitating and maintaining factors. These include identifying unhelpful patterns and making changes to activity and exercise patterns, sleep hygiene, thinking strategies, management of stress and emotions, and approaches to relationships and communication with others. BOOST aims to equip participants with a variety of appropriate cognitive, behavioral, and problem-solving skills so that they can continue to make further progress after the program is completed.

#### Content and Structure

The program comprises of 12 web-based sessions (8 core sessions and 4 symptom-specific sessions). An overview of the content covered in each session and the associated tasks is presented in Multimedia Appendix 1. Participants are advised to work through sessions, 1 at a time, at regular weekly intervals. The sessions are designed for participants to work through at their own pace, generally taking between 30 and 60 minutes to complete.

The order of the core sessions was drawn from the principles used in 2 of our previous successful digital CBT interventions for symptom management [19,65]. The first session focuses on education about contributing factors to IBD symptoms alongside self-assessment to provide patients with a coherent understanding of how cognitive behavioral approaches can help reduce symptom severity and impact. The next 2 sessions focus on behavioral strategies in the context of balancing activity patterns (eg, avoidance or all-or-nothing behavior) and sleep focus. These tasks are more concrete and easier for most patients to engage in at the start. Sessions 3 and 4 use cognitive techniques to identify challenging, unhelpful thoughts about symptoms and personal expectations. Cognitive techniques can be more challenging as they require meta-cognitive abilities. Our previous work indicated that it is helpful to include these once people are well-engaged in the program. The final core sessions on managing stress and social support incorporated both behavioral and cognitive methods drawing from the methods or techniques addressed in the previous 5 sessions. All sessions were tailored to IBD, including patient vignettes and IBD-specific examples. Symptom-specific sessions revisited the *vicious cycle* in relation to the symptoms and provided specific psychoeducation and techniques (eg, physiological factors related to fatigue, acute vs chronic pain, and bowel retraining exercises for urgency).

#### Features

The sessions are designed to be interactive, with personalized pathways tailored to a participant’s needs. Several features are included to facilitate participants’ completion of the intervention. These include a calendar where participants can input goals they are working toward, a virtual notepad where participants can write reflections on their progress, a goals section where they can review their weekly goals, a graphical representation of the impact of their symptoms on their daily activities over time, and personalized automated emails or SMS text messages reminding participants to log into the program. A paper copy of the intervention is also available, should participants request a PDF version.

#### Facilitator Support

The participants have access to a facilitator who supports them in their progress. Facilitators can manage their caseloads and see relevant patient information through the BOOST facilitator platform. All participants conduct a 30-minute phone call with their facilitator after completing the first session. In this initial call, the facilitator reviews the participant’s vicious cycle of symptoms with the participant to help them reflect on and clarify the factors they have identified as potentially contributing to their symptoms and guide the participant to set goals for the program. Facilitators follow a checklist to structure phone calls with participants (Multimedia Appendix 2) and send weekly SMS text messages to provide support and encouragement. For example, facilitators may help participants identify goals or prompt reflection following a session. The participants are reminded at the end of each session to seek extra support in progressing through the program by sending a web-based message to the facilitator.

#### Facilitator Training

Facilitators are required to complete training for BOOST, which entails attending training sessions from the research team, developing basic cognitive behavioral skills through role-plays, and practicing the telephone session with a *practice patient* (a volunteer with IBD; Multimedia Appendix 3). Facilitators are provided with a facilitator training manual and receive individual and group supervision to discuss patient cases and reflections with a BOOST supervisor (health psychologist).

### Modeling Processes and Outcomes: Feasibility and Acceptability Testing

Once the initial version of the web-based intervention was developed, 31 people with IBD (aged 18-65 years, female, 16/31, 52%, and with Crohn disease, 17/31, 55%) were given access to the full program for a period of 8 weeks and provided feedback on the program through web-based surveys or telephone calls after 1 (30/31, 97%), 4 (24/31, 77%), and 8 (21/31, 68%) weeks. First impressions from feasibility testing were positive, with participants commenting on the clean, bright, professional, and easy-to-use design and functionality of the website. Overall, sessions were rated as understandable, relevant, and having an appropriate tone and length and were recognized as helpful. Feedback was provided by participants following each session on how helpful, relevant, easy to navigate, and motivating they rated sessions (Multimedia Appendix 4). Setting and reviewing goals related to the content covered in the session were rated as easy to complete and useful. Participants often tracked the impact of their symptoms using the program’s symptom graph, which was rated as a useful tool for monitoring their symptoms over time. Although the tasks were rated as useful, the functionality and layout of the task page were identified as the areas that needed the most change. Therefore, additional usability testing was conducted with 13% (4/31) of the participants to obtain more detailed feedback on how to improve the page.

In addition, it was clear that certain features of the program were not used. Therefore, the visibility and accessibility of the vicious cycle, bookmarks, and notification settings were improved. There was no facilitator support during this testing phase; however, two-thirds felt that being able to contact a facilitator while working through the sessions would have been useful, and they would have been likely to contact them for reassurance, validation, and queries relating to the content. Overall, throughout the program, users were motivated to continue and were highly likely to recommend to a friend with IBD.

## Discussion

### Summary of Process

This paper describes the systematic application of theory, evidence, and stakeholder involvement in the development of BOOST, the first digital cognitive behavioral intervention, with the primary aim of lessening the impact of fatigue, pain, and urgency to improve the QoL of people with IBD. A review of the evidence suggests that the cognitive behavioral model of symptom perpetuation provides a valid framework for the intervention. A mapping of findings across empirical studies looking at cognitive behavioral correlates of pain, fatigue, or urgency in IBD identified core transsymptom factors to be targeted in the intervention, including creating consistent daily routines or activity patterns, regulating sleep, and identifying and challenging unhelpful thoughts about symptoms. Symptom-specific factors such as the role of practical exercises for managing urgency [16], acceptance of chronic pain [56], and fatigue-specific beliefs [60], were also identified. Content and therapeutic techniques were mapped to create an intervention logic model. A 12-session (8 transsymptom- and 4 symptom-specific) tailored, interactive web program was then built with patient and nurse facilitator dashboards. Stakeholder input from 87 people with IBD and 60 nurses informed the build throughout, including the content and features of the website. A feasibility study with 31 people with IBD confirmed that the acceptability of the program was high and suggested further modifications of some features and language, which were made.

The intervention’s theory-based logic model provides a rigorous and transparent summary of the intervention processes and mechanisms, where identified modifiable psychosocial factors were mapped onto cognitive behavioral techniques to influence intervention outcomes. Logic models provided a sophisticated way of communicating program theory to both stakeholders and the research team, facilitated process and outcome evaluation in a randomized controlled trial (RCT) [70,71], and are recommended in the 2019 MRC guidelines. Although this guidance was published after the intervention development started, the actions recommended by O’Cathain et al [71] were each addressed because of the complementary nature of the intervention approaches used (Multimedia Appendix 5).

The development of the intervention with stakeholders allowed the design team to ensure that an acceptable, personalized, and interactive intervention was developed. These features are recognized as essential components of self-guided web-based interventions to improve user experience and clinical outcomes in the context of chronic physical health conditions [72]. Feedback on sessions and tasks was positive, with individuals describing the content as relevant, understandable, and helpful. Suggestions for improvement led to key modifications to the intervention, such as the use of a graphical symptom tracker and a personal calendar to log goals. Feasibility testing of the intervention provided an opportunity to understand how relevant and user-friendly the content and intervention features were, respectively, and identify any limitations apparent to users. This subsequently led to further changes to improve the visibility and accessibility of website functions, reinforcing the iterative nature of development and evaluation.

Understanding users’ needs (referring to both recipients and deliverers of the intervention), as recommended by the person-based approach and NPT, provided an opportunity to learn about the crucial contextual factors influencing intervention delivery. Focus groups revealed the limited time available to nurses to support the intervention, their preference for predominantly SMS text messaging or email communication, and the need for comprehensive training and supervision. They subsequently informed the intensity and modality of the facilitator support and training program. Understanding the day-to-day practices of IBD nurses was fundamental to optimizing sustainability and the likelihood of the intervention being adopted [73]. The provision of adequate training to support health care professionals in integrating web-based interventions into day-to-day practice has been emphasized elsewhere [74].

### Conclusions

The development of complex health interventions evaluated in RCTs has often lacked transparent reporting of the development process. This paper describes the development of a digital, tailored, facilitator-supported self-management intervention based on a cognitive behavioral model of symptom perpetuation for patients with IBD and symptoms of fatigue, pain, and urgency/incontinence. It presents the integration of intervention development frameworks and how these were used to inform the use of theory, empirical evidence, and stakeholder input to develop a transsymptomatic intervention, with the aim of improving QoL and reducing symptom burden.

The lack of RCTs describing how their interventions were developed has been highlighted previously [75]. This paper provides a robust and transparent description of the development of a web-based intervention for symptoms of fatigue, pain, and urgency in IBD, with the aim of improving QoL and reducing symptom burden. This demonstrates the compatibility of combining the MRC framework and person-based approach, where evidence was identified, the theory was developed, mechanisms and processes were outlined, and user-centered feedback informed the intervention content and functionality. This comprehensive and iterative approach to intervention development is argued to facilitate the effectiveness and efficacy of a complex health intervention and its long-term implementation [76]. BOOST is now being tested in a National Institute for Health Research–funded large-scale RCT [42].

## Appendix

Multimedia Appendix 1 IBD-BOOST final intervention sessions and tasks.

Multimedia Appendix 2 Facilitator checklist for telephone sessions.

Multimedia Appendix 3 IBD-BOOST facilitator training outline.

Multimedia Appendix 4 Mean scores for session feedback. Likert scale (0-5) assessing how helpful, relevant, easy to navigate, and motivating sessions were to participants taking part in feasibility testing of the intervention.

Multimedia Appendix 5 Actions to consider in intervention development in updated Medical Research Council guidance [71] and descriptions of how these were addressed in BOOST.

## Abbreviations

CBT: cognitive behavioral therapy
IBD: inflammatory bowel disease
IBS: irritable bowel syndrome
MRC: Medical Research Council
NIHR: National Institute for Health Research
NPT: normalization process theory
PPI: patient and public involvement
QoL: quality of life
RCT: randomized controlled trial

## References

[1] Molodecky NA, Soon IS, Rabi DM, Ghali WA, Ferris M, Chernoff G, Benchimol EI, Panaccione R, Ghosh S, Barkema HW, Kaplan GG (2012). Increasing incidence and prevalence of the inflammatory bowel diseases with time, based on systematic review. Gastroenterology.

[2] Ng SC, Shi HY, Hamidi N, Underwood FE, Tang W, Benchimol EI, Panaccione R, Ghosh S, Wu JC, Chan FK, Sung JJ, Kaplan GG (2017). Worldwide incidence and prevalence of inflammatory bowel disease in the 21st century: a systematic review of population-based studies. Lancet.

[3] Pillai N, Dusheiko M, Burnand B, Pittet V (2017). A systematic review of cost-effectiveness studies comparing conventional, biological and surgical interventions for inflammatory bowel disease. PLoS One.

[4] Burisch J, Kiudelis G, Kupcinskas L, Kievit HA, Andersen KW, Andersen V, Salupere R, Pedersen N, Kjeldsen J, D'Incà R, Valpiani D, Schwartz D, Odes S, Olsen J, Nielsen KR, Vegh Z, Lakatos PL, Toca A, Turcan S, Katsanos KH, Christodoulou DK, Fumery M, Gower-Rousseau C, Zammit SC, Ellul P, Eriksson C, Halfvarson J, Magro FJ, Duricova D, Bortlik M, Fernandez A, Hernández V, Myers S, Sebastian S, Oksanen P, Collin P, Goldis A, Misra R, Arebi N, Kaimakliotis IP, Nikuina I, Belousova E, Brinar M, Cukovic-Cavka S, Langholz E, Munkholm P, Epi-IBD group (2019). Natural disease course of Crohn's disease during the first 5 years after diagnosis in a European population-based inception cohort: an Epi-IBD study. Gut.

[5] Farrell D, McCarthy G, Savage E (2016). Self-reported symptom burden in individuals with inflammatory bowel disease. J Crohns Colitis.

[6] Czuber-Dochan W, Ream E, Norton C (2013). Review article: description and management of fatigue in inflammatory bowel disease. Aliment Pharmacol Ther.

[7] Artom M, Czuber-Dochan W, Sturt J, Norton C (2016). Targets for health interventions for inflammatory bowel disease-fatigue. J Crohns Colitis.

[8] Norton C, Dibley LB, Bassett P (2013). Faecal incontinence in inflammatory bowel disease: associations and effect on quality of life. J Crohns Colitis.

[9] Lönnfors S, Vermeire S, Greco M, Hommes D, Bell C, Avedano L (2014). IBD and health-related quality of life -- discovering the true impact. J Crohns Colitis.

[10] Hart AL, Lomer M, Verjee A, Kemp K, Faiz O, Daly A, Solomon J, McLaughlin J (2017). What are the top 10 research questions in the treatment of inflammatory bowel disease? A priority setting partnership with the James Lind Alliance. J Crohns Colitis.

[11] Elkjaer M, Moser G, Reinisch W, Durovicova D, Lukas M, Vucelic B, Wewer V, Frederic Colombel J, Shuhaibar M, O'Morain C, Politi P, Odes S, Bernklev T, Oresland T, Nikulina I, Belousova E, Van der Eijk I, Munkholm P (2008). IBD patients need in health quality of care ECCO consensus. J Crohns Colitis.

[12] Grover M, Herfarth H, Drossman DA (2009). The functional-organic dichotomy: postinfectious irritable bowel syndrome and inflammatory bowel disease-irritable bowel syndrome. Clin Gastroenterol Hepatol.

[13] Halpin SJ, Ford AC (2012). Prevalence of symptoms meeting criteria for irritable bowel syndrome in inflammatory bowel disease: systematic review and meta-analysis. Am J Gastroenterol.

[14] Colombel JF, Shin A, Gibson PR (2019). AGA clinical practice update on functional gastrointestinal symptoms in patients with inflammatory bowel disease: expert review. Clin Gastroenterol Hepatol.

[15] Sweeney L, Moss-Morris R, Czuber-Dochan W, Meade L, Chumbley G, Norton C (2018). Systematic review: psychosocial factors associated with pain in inflammatory bowel disease. Aliment Pharmacol Ther.

[16] Proudfoot H, Norton C, Artom M, Didymus E, Kubasiewicz S, Khoshaba B (2018). Targets for interventions for faecal incontinence in inflammatory bowel disease: a systematic review. Scand J Gastroenterol.

[17] Stanisic V, Quigley EM (2014). The overlap between IBS and IBD: what is it and what does it mean?. Expert Rev Gastroenterol Hepatol.

[18] Windgassen S, Moss-Morris R, Chilcot J, Sibelli A, Goldsmith K, Chalder T (2017). The journey between brain and gut: a systematic review of psychological mechanisms of treatment effect in irritable bowel syndrome. Br J Health Psychol.

[19] Everitt HA, Landau S, O'Reilly G, Sibelli A, Hughes S, Windgassen S, Holland R, Little P, McCrone P, Bishop F, Goldsmith K, Coleman N, Logan R, Chalder T, Moss-Morris R, ACTIB trial group (2019). Assessing telephone-delivered cognitive-behavioural therapy (CBT) and web-delivered CBT versus treatment as usual in irritable bowel syndrome (ACTIB): a multicentre randomised trial. Gut.

[20] Ford AC, Lacy BE, Harris LA, Quigley EM, Moayyedi P (2019). Effect of antidepressants and psychological therapies in irritable bowel syndrome: an updated systematic review and meta-analysis. Am J Gastroenterol.

[21] Artom M, Czuber-Dochan W, Sturt J, Proudfoot H, Roberts D, Norton C (2019). Cognitive-behavioural therapy for the management of inflammatory bowel disease-fatigue: a feasibility randomised controlled trial. Pilot Feasibility Stud.

[22] Sweeney L, Moss-Morris R, Czuber-Dochan W, Norton C (2021). Pain management in inflammatory bowel disease: feasibility of an online therapist-supported CBT-based self-management intervention. Pilot Feasibility Stud.

[23] McCombie AM, Mulder RT, Gearry RB (2013). Psychotherapy for inflammatory bowel disease: a review and update. J Crohns Colitis.

[24] Ballou S, Keefer L (2017). Psychological interventions for irritable bowel syndrome and inflammatory bowel diseases. Clin Transl Gastroenterol.

[25] Bossuyt P, Pouillon L, Bonnaud G, Danese S, Peyrin-Biroulet L (2017). E-health in inflammatory bowel diseases: more challenges than opportunities?. Dig Liver Dis.

[26] Yardley L, Morrison LG, Andreou P, Joseph J, Little P (2010). Understanding reactions to an internet-delivered health-care intervention: accommodating user preferences for information provision. BMC Med Inform Decis Mak.

[27] Jackson BD, Gray K, Knowles SR, De Cruz P (2016). EHealth technologies in inflammatory bowel disease: a systematic review. J Crohns Colitis.

[28] Kemp K, Dibley L, Chauhan U, Greveson K, Jäghult S, Ashton K, Buckton S, Duncan J, Hartmann P, Ipenburg N, Moortgat L, Theeuwen R, Verwey M, Younge L, Sturm A, Bager P (2018). Second N-ECCO consensus statements on the European nursing roles in caring for patients with Crohn's disease or ulcerative colitis. J Crohns Colitis.

[29] Hanlon I, Hewitt C, Bell K, Phillips A, Mikocka-Walus A (2018). Systematic review with meta-analysis: online psychological interventions for mental and physical health outcomes in gastrointestinal disorders including irritable bowel syndrome and inflammatory bowel disease. Aliment Pharmacol Ther.

[30] O'Cathain A, Croot L, Sworn K, Duncan E, Rousseau N, Turner K, Yardley L, Hoddinott P (2019). Taxonomy of approaches to developing interventions to improve health: a systematic methods overview. Pilot Feasibility Stud.

[31] Craig P, Dieppe P, Macintyre S, Michie S, Nazareth I, Petticrew M, Medical Research Council Guidance (2008). Developing and evaluating complex interventions: the new Medical Research Council guidance. BMJ.

[32] Yardley L, Morrison L, Bradbury K, Muller I (2015). The person-based approach to intervention development: application to digital health-related behavior change interventions. J Med Internet Res.

[33] May C, Finch T (2009). Implementing, embedding, and integrating practices: an outline of normalization process theory. Sociology.

[34] May CR, Mair F, Finch T, MacFarlane A, Dowrick C, Treweek S, Rapley T, Ballini L, Ong BN, Rogers A, Murray E, Elwyn G, Légaré F, Gunn J, Montori VM (2009). Development of a theory of implementation and integration: Normalization Process Theory. Implement Sci.

[35] Jackson BD, Con D, De Cruz P (2018). Design considerations for an eHealth decision support tool in inflammatory bowel disease self-management. Intern Med J.

[36] Simblett S, Greer B, Matcham F, Curtis H, Polhemus A, Ferrão J, Gamble P, Wykes T (2018). Barriers to and facilitators of engagement with remote measurement technology for managing health: systematic review and content analysis of findings. J Med Internet Res.

[37] Hudson JL, Moon Z, Hughes LD, Moss-Morris R, Hagger MS, Cameron LD, Hamilton K, Hankonen N, Lintunen T (2020). 24 Engagement of stakeholders in the design, evaluation, and implementation of complex interventions. The handbook of behavior change.

[38] Dibley L, Khoshaba B, Artom M, Van Loo V, Sweeney L, Syred J, Windgassen S, Moffatt G, Norton C, members of the IBD-BOOST PPI team (2021). Patient strategies for managing the vicious cycle of fatigue, pain and urgency in inflammatory bowel disease: impact, planning and support. Dig Dis Sci.

[39] Fawson S, Dibley L, Smith K, Batista J, Artom M, Windgassen S, Syred J, Moss-Morris R, Norton C (2021). Developing an online program for self-management of fatigue, pain, and urgency in inflammatory bowel disease: patients' needs and wants. Dig Dis Sci.

[40] van den Haak MJ, de Jong MD, Schellens PJ (2007). Evaluation of an informational web site: three variants of the think-aloud method compared. Tech Commun.

[41] Hardeman W, Sutton S, Griffin S, Johnston M, White A, Wareham NJ, Kinmonth AL (2005). A causal modelling approach to the development of theory-based behaviour change programmes for trial evaluation. Health Educ Res.

[42] Norton C, Syred J, Kerry S, Artom M, Sweeney L, Hart A, Czuber-Dochan W, Taylor SJ, Mihaylova B, Roukas C, Aziz Q, Miller L, Pollok R, Saxena S, Stagg I, Terry H, Zenasni Z, Dibley L, Moss-Morris R (2021). Supported online self-management versus care as usual for symptoms of fatigue, pain and urgency/incontinence in adults with inflammatory bowel disease (IBD-BOOST): study protocol for a randomised controlled trial. Trials.

[43] Branch JL (2000). Investigating the information-seeking processes of adolescents - the value of using think alouds and think afters. Libr Inf Sci Res.

[44] Farrell D, Artom M, Czuber-Dochan W, Jelsness-Jørgensen LP, Norton C, Savage E (2020). Interventions for fatigue in inflammatory bowel disease. Cochrane Database Syst Rev.

[45] Norton C, Czuber-Dochan W, Artom M, Sweeney L, Hart A (2017). Systematic review: interventions for abdominal pain management in inflammatory bowel disease. Aliment Pharmacol Ther.

[46] Nigam GB, Limdi JK, Vasant DH (2018). Current perspectives on the diagnosis and management of functional anorectal disorders in patients with inflammatory bowel disease. Therap Adv Gastroenterol.

[47] Beck AT (1964). Thinking and depression. II. Theory and therapy. Arch Gen Psychiatry.

[48] Turk DC, Meichenbaum D, Genest M (1983). Pain and behavioral medicine: a cognitive-behavioral perspective.

[49] Deary V, Chalder T, Sharpe M (2007). The cognitive behavioural model of medically unexplained symptoms: a theoretical and empirical review. Clin Psychol Rev.

[50] Norton C, Czuber-Dochan W, Bassett P, Berliner S, Bredin F, Darvell M, Forbes A, Gay M, Ream E, Terry H (2015). Assessing fatigue in inflammatory bowel disease: comparison of three fatigue scales. Aliment Pharmacol Ther.

[51] Ratnakumaran R, Warren L, Gracie DJ, Sagar RC, Hamlin PJ, O'Connor A, Ford AC (2018). Fatigue in inflammatory bowel disease reflects mood and symptom-reporting behavior rather than biochemical activity or anemia. Clin Gastroenterol Hepatol.

[52] Artom M, Czuber-Dochan W, Sturt J, Norton C (2017). Cognitive behavioural therapy for the management of inflammatory bowel disease-fatigue with a nested qualitative element: study protocol for a randomised controlled trial. Trials.

[53] Sweeney L, Moss-Morris R, Czuber-Dochan W, Murrells T, Norton C (2020). Developing a better biopsychosocial understanding of pain in inflammatory bowel disease: a cross-sectional study. Eur J Gastroenterol Hepatol.

[54] Dibley L, Norton C (2013). Experiences of fecal incontinence in people with inflammatory bowel disease: self-reported experiences among a community sample. Inflamm Bowel Dis.

[55] Czuber-Dochan W, Dibley LB, Terry H, Ream E, Norton C (2013). The experience of fatigue in people with inflammatory bowel disease: an exploratory study. J Adv Nurs.

[56] Sweeney L, Moss-Morris R, Czuber-Dochan W, Belotti L, Kabeli Z, Norton C (2019). 'It's about willpower in the end. You've got to keep going': a qualitative study exploring the experience of pain in inflammatory bowel disease. Br J Pain.

[57] Dibley L, Norton C, Cotterill N, Bassett P (2016). Development and initial validation of a disease-specific bowel continence questionnaire for inflammatory bowel disease patients: the ICIQ-IBD. Eur J Gastroenterol Hepatol.

[58] Salkovskis PM, Gregory JD, Sedgwick-Taylor A, White J, Opher S, Ólafsdóttir S (2016). Extending cognitive-behavioural theory and therapy to medically unexplained symptoms and long-term physical conditions: a hybrid transdiagnostic/problem specific approach. Behav Change.

[59] Hulme K, Hudson JL, Rojczyk P, Little P, Moss-Morris R (2017). Biopsychosocial risk factors of persistent fatigue after acute infection: a systematic review to inform interventions. J Psychosom Res.

[60] Artom M, Czuber-Dochan W, Sturt J, Murrells T, Norton C (2017). The contribution of clinical and psychosocial factors to fatigue in 182 patients with inflammatory bowel disease: a cross-sectional study. Aliment Pharmacol Ther.

[61] Keogh A, Burke M (2017). Faecal incontinence, anxiety and depression in inflammatory bowel disease. Gastrointest Nurs.

[62] Hall NJ, Rubin GP, Dougall A, Hungin AP, Neely J (2005). The fight for 'health-related normality': a qualitative study of the experiences of individuals living with established inflammatory bowel disease (ibd). J Health Psychol.

[63] Gromisch ES, Kerns RD, Czlapinski R, Beenken B, Otis J, Lo AC, Beauvais J (2020). Cognitive behavioral therapy for the management of multiple sclerosis-related pain: a randomized clinical trial. Int J MS Care.

[64] Uebelacker LA, Weisberg RB, Herman DS, Bailey GL, Pinkston-Camp MM, Garnaat SL, Stein MD (2016). Pilot randomized trial of collaborative behavioral treatment for chronic pain and depression in persons living with HIV/AIDS. AIDS Behav.

[65] Moss-Morris R, McCrone P, Yardley L, van Kessel K, Wills G, Dennison L (2012). A pilot randomised controlled trial of an Internet-based cognitive behavioural therapy self-management programme (MS Invigor8) for multiple sclerosis fatigue. Behav Res Ther.

[66] Menees SB, Almario CV, Spiegel BM, Chey WD (2018). Prevalence of and factors associated with fecal incontinence: results from a population-based survey. Gastroenterology.

[67] Hunt MG, Wong C, Aajmain S, Dawodu I (2018). Fecal incontinence in people with self-reported irritable bowel syndrome: prevalence and quality of life. J Psychosom Res.

[68] Norton C, Kamm MA (2001). Anal sphincter biofeedback and pelvic floor exercises for faecal incontinence in adults--a systematic review. Aliment Pharmacol Ther.

[69] Matthias C, Fawson S, Yan L, Sweeney L, Moss-Morris R, Norton C (2021). Inflammatory bowel disease nurses' views on taking on a new role to support an online self-management programme for symptoms of fatigue, pain and urgency: a qualitative study to maximise intervention acceptance. Gastrointest Nurs.

[70] Moore GF, Audrey S, Barker M, Bond L, Bonell C, Hardeman W, Moore L, O'Cathain A, Tinati T, Wight D, Baird J (2015). Process evaluation of complex interventions: Medical Research Council guidance. BMJ.

[71] O'Cathain A, Croot L, Duncan E, Rousseau N, Sworn K, Turner KM, Yardley L, Hoddinott P (2019). Guidance on how to develop complex interventions to improve health and healthcare. BMJ Open.

[72] Xie LF, Itzkovitz A, Roy-Fleming A, Da Costa D, Brazeau AS (2020). Understanding self-guided web-based educational interventions for patients with chronic health conditions: systematic review of intervention features and adherence. J Med Internet Res.

[73] Hallberg IR, Richards DA, Richards DA, Hallberg IR (2015). A few final thoughts. Complex interventions in health: an overview of research methods.

[74] Davies F, Shepherd HL, Beatty L, Clark B, Butow P, Shaw J (2020). Implementing Web-based therapy in routine mental health care: systematic review of health professionals' perspectives. J Med Internet Res.

[75] Kwasnicka D, Dombrowski SU, White M, Sniehotta F (2016). Theoretical explanations for maintenance of behaviour change: a systematic review of behaviour theories. Health Psychol Rev.

[76] Bleijenberg N, de Man-van Ginkel JM, Trappenburg JC, Ettema RG, Sino CG, Heim N, Hafsteindóttir TB, Richards DA, Schuurmans MJ (2018). Increasing value and reducing waste by optimizing the development of complex interventions: enriching the development phase of the Medical Research Council (MRC) Framework. Int J Nurs Stud.

