# Supplementary material for: A Novel Digital Self-management Intervention for Symptoms of Fatigue, Pain, and Urgency in Inflammatory Bowel Disease: Describing the Process of Development
Source: JMIR Form Res. 2022 May 18;6(5):e33001. doi: 10.2196/33001 (PMC9161057; doi:10.2196/33001)
Supplement: Multimedia Appendix 1 [file formative_v6i5e33001_app1.docx]

**Multimedia Appendix 1 -** IBD-BOOST Final Intervention Sessions & Tasks

| Session Number and Topic | Description of Content |
| --- | --- |
| Session 1. Understanding your IBD symptoms | Factors that can contribute to fatigue, pain and urgency in IBD. Identifying specific factors and developing a personal vicious cycle of symptoms. Use of self-monitoring not symptom focusing. Setting aims for the programme.  *Task: Symptom severity and stress level diary.* |
| Session 2. Balancing your activity, eating and exercise | Importance of activity and exercise. How fear leads to avoidance. Eating patterns. Setting goals for activity and exercise.  *Task: Reviewing and working toward goals for activity + sleep diary.* |
| Session 3. Improving your Sleep | Why is sleep important? Sleep patterns & habits. Improving sleep.  Setting goals for sleep.  *Task: Reviewing and working toward goals for sleeping patterns and habits.* |
| Session 4a. Changing your thoughts: Part 1 | Why are thoughts important? Identifying unhelpful thinking.  *Task: Thought record.* |
| Session 4b. Changing your thoughts: Part 2 | Developing alternative thoughts.  *Task: Alternative thought record.* |
| Session 5. Managing stress and coping with emotions | The effects of stress and finding ways to manage it. The role of emotions and determining how best to take care of oneself. Setting goals for managing stress and emotions.  *Task: Reviewing and working toward goals for stress management + stress diary* |
| Session 6. Making the most of your social support and communication | Types of social support. Communication and disclosure. Setting goals for social support.  *Task: Reviewing and working toward goals for social support.* |
| Session 7. Managing and understanding fatigue in IBD | Types of fatigue. Factors related to IBD fatigue. Exploring a vicious cycle of IBD fatigue; the role of thoughts, emotions and behaviours. |
| Session 8. Managing and understanding pain in IBD | Difference between acute and chronic pain in IBD. Factors related to IBD-pain. Exploring a vicious cycle of IBD pain; the role of thoughts, emotions and behaviours. Common questions around pain in IBD. |
| Session 9. Managing urgency and leakage | Bowel functioning and bowel control difficulties. Stress and anxiety in urgency. Exercises to help reduce accidents. Practical bowel management tips. Using social networks to help manage urgency. |
| Session 10. The role of acceptance and self-compassion in pain | What is acceptance and how can it help? The role of resilience. Practical exercises. |
| Session 11. Summary and maintaining improvement | Reviewing programme aims. Preparing for the future. Sustaining and building upon improvements. |
