# Supplementary material for: A Novel Digital Self-management Intervention for Symptoms of Fatigue, Pain, and Urgency in Inflammatory Bowel Disease: Describing the Process of Development
Source: JMIR Form Res. 2022 May 18;6(5):e33001. doi: 10.2196/33001 (PMC9161057; doi:10.2196/33001)
Supplement: Multimedia Appendix 2 [file formative_v6i5e33001_app2.docx]

**Multimedia Appendix 2 -** Facilitator checklist for telephone sessions

| **Task Type** | **Task** | **Completion** |
| --- | --- | --- |
| **Introduction to session** | Introductions |  |
|  | Check practicalities and technical issues |  |
|  | Set an agenda |  |
| **Assessment** | Symptoms |  |
|  | Severity and impact of symptoms on daily activities |  |
|  | Triggers |  |
| **CBT model** | Presentation of CBT model |  |
|  | Going through personal vicious cycle |  |
|  | Setting direction for the programme |  |
| **Programme aims** | Programme aims in relation to the content |  |
| **Closing the session** | Additional questions |  |
