# Supplementary material for: A Novel Digital Self-management Intervention for Symptoms of Fatigue, Pain, and Urgency in Inflammatory Bowel Disease: Describing the Process of Development
Source: JMIR Form Res. 2022 May 18;6(5):e33001. doi: 10.2196/33001 (PMC9161057; doi:10.2196/33001)
Supplement: Multimedia Appendix 3 [file formative_v6i5e33001_app3.docx]

**Multimedia Appendix 3 –** IBD-BOOST Facilitator Training Outline

| **Stage of Training** | **Overview** | **Details** |
| --- | --- | --- |
| **Stage 1** | Reading Materials | Facilitator Training Manual provided prior to training. |
| **Stage 2** | Overview of BOOST RCT  Introduction to CBT | 1-hour training session: Interactive presentation + exercises. |
| **Stage 3** | Navigating the BOOST website | 1-hour training session: Interactive presentation + exercises. |
| **Stage 4** | Facilitator-Patient Contact  & CBT Skills Training  (part 1) | 1-hour training session: Interactive presentation + exercise. Therapeutic skills and role plays. Homework practice of role play using training materials. |
| **Stage 5** | Facilitator-Patient Contact  & CBT Skills Training  (part 2) | 1-hour training session: In-site messaging. Therapeutic skills and role plays. Homework practice of role play using training materials. |
| **Practice Patient** | Practice patient exercise  Supervision | 30-minute audio recorded phone call and in-site messages with volunteer with IBD. Competencies during phone call assessed by supervisor: guided discovery ii) empathy and sensitivity iii) validation. |
| **Supervision** | Individual and Group Supervision | Combination of individual and group supervision (fortnightly/monthly) with supervisor to review patient cases and phone call/messaging queries. |
