# Supplementary material for: A Novel Digital Self-management Intervention for Symptoms of Fatigue, Pain, and Urgency in Inflammatory Bowel Disease: Describing the Process of Development
Source: JMIR Form Res. 2022 May 18;6(5):e33001. doi: 10.2196/33001 (PMC9161057; doi:10.2196/33001)
Supplement: Multimedia Appendix 4 [file formative_v6i5e33001_app4.docx]

**Multimedia Appendix 4.** Mean scores for session feedback. Likert scale [0-5] assessing how helpful, relevant, easy to navigate and motivating sessions were to participants taking part in feasibility testing of the intervention. NB – only three participants provided feedback on the pain session of which one said it wasn’t relevant to them as they didn’t have pain.
