# Supplementary material for: A Novel Digital Self-management Intervention for Symptoms of Fatigue, Pain, and Urgency in Inflammatory Bowel Disease: Describing the Process of Development
Source: JMIR Form Res. 2022 May 18;6(5):e33001. doi: 10.2196/33001 (PMC9161057; doi:10.2196/33001)
Supplement: Multimedia Appendix 5 [file formative_v6i5e33001_app5.docx]

**Multimedia Appendix 5.** Actions to consider in intervention development in updated MRC guidance {O'Cathain, 2019 #86}[71] and descriptions of how these were addressed in BOOST

| **Actions to consider**  **[O’Cathain et al. 2019]** | **Actions carried out in BOOST intervention development** |
| --- | --- |
| Plan the process | Several publications on impact and psychosocial factors associated with fatigue, pain and urgency. |
| Involve stakeholders | 87 people with IBD and 60 nurses + experts in gastroenterology, health psychology and nursing |
| Bring together a team | Intervention development team comprised health psychologists [lead], nurses, people with IBD |
| Reviewed published evidence | Identified evidence for fatigue, pain and urgency including systematic reviews and empirical studies |
| Draw on existing theories | Applied theory that has shown an evidence-base in symptom management in other long term conditions |
| Articulate programme theory | Developed an intervention logic model. Identified overlapping and distinct psychosocial factors |
| Undertake primary data collection | Quantitative and qualitative research conducted to explore psychosocial factors associated with symptoms of fatigue, pain and urgency |
| Understand context | Focus groups with IBD nurses to understand feasibility of provision of facilitation. People with IBD and IBD healthcare professionals embedded in intervention development team |
| Attend to future implementation | Focus groups with IBD nurses to understand feasibility around provision of support |
| Design and refine | Modifications to design/content/functionalities following user-centred feedback |
